# Editorial: Advances in deep learning methods for medical image analysis

**DOI:** 10.3389/fradi.2022.1097533

**Published:** 2023-01-06

**Authors:** Heung-Il Suk, Mingxia Liu, Xiaohuan Cao, Jaeil Kim

**Affiliations:** ^1^ Department of Artificial Intelligence, Korea University, Seoul, South Korea; ^2^ Department of Brain and Cognitive Engineering, Korea University, Seoul, South Korea; ^3^ Department of Radiology and BRIC, University of North Carolina at Chapel Hill, Chapel Hill, NC, United States; ^4^ Shanghai United Imaging Intelligence Co., Ltd., Shanghai, China; ^5^ School of Computer Science & Engineering, Kyungpook National University, Daegu, South Korea

**Keywords:** editorial, deep learning, artificial intelligence, medical image analysis, neural network

**Editorial on the Research Topic** Advances in deep learning methods for medical image analysis

The rapid development of artificial intelligence (AI) technology is leading many innovations in the medical field and is playing a major role in establishing objective, consistent, and efficient medical environments with large-scale data. Deep learning represented by convolutional neural networks has achieved remarkable performance improvement in medical image processing fields such as image segmentation, registration, and enhancement. Furthermore, AI technology with deep learning is pioneering medical applications, such as lesion detection, differential diagnosis, disease prognosis, and surgical planning. More advanced AI technologies, such as transformers with self-attention mechanisms, allowing for learning global dependencies, have been widely applied, which further enhanced the capability of deep learning to analyze medical images. However, despite the remarkable advances in deep learning, many challenges remain. For example, when training data are biased or incomplete, deep learning models may fail to achieve the good generalization capability required to solve real-world problems. In addition, the limitations of deep learning models in interpreting results, and misunderstandings of their intended uses and hypotheses make it difficult for AI to gain trust in healthcare settings. In this regard, disease-specific neural networks, generalized learning methods, high-quality training data, and external evaluation based on testable hypotheses can ensure the reliability of medical AI technologies for humans (1, 2).

In medical image analysis, image segmentation has a role to accurately delineate organs or lesions from medical images, and to provide quantitative information of target objects. It is important for image segmentation to understand the various patterns of target objects in medical images, and to show robust performance against large variations in image quality. In this special issue on “Advances in Deep Learning Methods for Medical Image Analysis”, Zhang et al. proposed a brain tumor segmentation approach that ensembles three segmentation networks based on U-Net that receive different MR modalities (such as T1-weighted, T2-weighted and brain parcellation) as input, and also used a post-processing strategy to distinguish small enhanced tumors to reduce false-positives Zhang et al. Kuijawa et al. proposed ensemble-based and multi-task neural networks to perform Koos grading for Vestibular Schwannoma (VS) using brain tumor segmentation Kuijawa et al. In particular, the performance of Koos grading was improved by majority voting between an end-to-end neural network with multi-modal MR images and random forest models using radiomic features extracted from the segmentation of the brain structures related to VS Kuijawa et al. Bouget et al. showed the ability of U-Net-based architecture with an attention mechanism in segmenting Meningiomas (a type of brain tumor) with 98% accuracy for brain lesions larger than 3 ml Bouget et al. Aboutalebi et al. introduced a multi-scale encoder-decoder self-attention mechanism tailored for medical image analysis. They achieved higher performance in identifying SARS-CoV-2 in chest x-rays than conventional attention-based U-Net architectures Aboutalebi et al. These studies show that neural network architectures can improve generalization by learning and discovering varied disease-related pattern representations in medical images. At the same time, the fusion of multiple models helps provide diverse information required in the diagnostic process.

Although there is no golden method that can solve all the challenges faced in natural environments, it seems evident that deep learning technology is augmenting human ability in medical image analysis and raising new approaches to diagnosis and treatment in clinical settings. In the next few years, we may observe even more rapid changes in the medical environment led by AI with deep learning, further requiring more reliable AI technologies. Researchers will continue to investigate advanced AI technologies, just as transformers are now widely applied to the medical imaging field, and more advanced algorithms are expected.
